# Interaction between gut microbiota and toll-like receptor: from immunity to metabolism

**DOI:** 10.1007/s00109-016-1474-4

**Published:** 2016-09-17

**Authors:** Jensen H.C. Yiu, Bernhard Dorweiler, Connie W. Woo

**Affiliations:** 1 The State Key Laboratory of Pharmaceutical Biotechnology, Hong Kong, SAR China; 2 Department of Pharmacology and Pharmacy, the University of Hong Kong, Hong Kong, SAR China; 3 Division of Vascular Surgery, Department of Cardiothoracic & Vascular Surgery, University Medical Center, Mainz, Germany

**Keywords:** Toll-like receptor, Gut microbiota, Metabolic regulation

## Abstract

The human gut contains trillions of commensal bacteria, and similar to pathogenic bacteria, the gut microbes and their products can be recognized by toll-like receptors (TLRs). It is well acknowledged that the interaction between gut microbiota and the local TLRs help to maintain the homeostasis of intestinal immunity. High-fat intake or obesity can weaken gut integrity leading to the penetration of gut microbiota or their bacterial products into the circulation, leading to the activation of TLRs on immune cells and subsequently low-grade systemic inflammation in host. Metabolic cells including hepatocytes and adipocytes also express TLRs. Although they are able to produce and secrete inflammatory molecules, the effectiveness remains low compared with the immune cells embedded in the liver and adipose tissue. The interaction of TLRs in these metabolic cells or organs with gut microbiota remains unclear, but a few studies have suggested that the functions of these TLRs are related to metabolism. Alteration of the gut microbiota is associated with body weight change and adiposity in human, and the interaction between the commensal gut microbiota and TLRs may possibly involve both metabolic and immunological regulation. In this review, we will summarize the current findings on the relationship between TLRs and gut microbiota with a focus on metabolic regulation and discuss how such interaction participates in host metabolism.

## Introduction

Trillions of commensal bacteria reside in our gastrointestinal tract, and the interactions between gut microbiota and the toll-like receptors (TLRs) on intestinal epithelial cells and immune cells help to maintain the homeostasis of our immune system [1]. TLRs are also expressed in hepatocytes and adipocytes. Although they are able to produce inflammatory molecules, the effectiveness remains low compared with the immune cells embedded in the liver and adipose tissue, rendering the function of TLRs in these cells elusive [2–4]. A few studies suggest that their function is metabolism-related [5]. Several global knockout models of TLRs or related pathways including TLR2, TLR5, interferon regulatory factor-3 (IRF3), and IRF5 which represent defects in immunity show an increase in body weight or fat mass regardless of other metabolic phenotypes [6–9]. The cell-specific knockout models also show the metabolic link, but the phenotypes are rather diverse. For example, hepatocyte-specific TLR4-knockout model shows an improvement in overall metabolic phenotypes upon high-fat diet challenge, but conversely, the same specific TLR5-knockout model displays an opposite phenotype including increased body weight, fatty liver, and fasting blood glucose [10, 11]. Inflammation mediated by TLR activation leads to downregulation of metabolism-related genes in the adipose tissue and liver [12]. Low-grade inflammation is often observed in obesity and metabolic diseases due to the increased gut permeability, and presumably, the penetration of molecules produced by gut microbiota can activate peripheral TLRs [13]. One of the functions of TLR pathway is to regulate intrinsic metabolism in immune cells in order to spare the energy for immune response [14]. Whether such energy relocation happens at an intercellular level or even cross-organ level remains unknown. The function of TLRs in innate and adaptive immunity and how TLRs modulate host immunity via the interaction with gut microbiota are reviewed elsewhere [15]. In this review, we mainly focus on the current findings of the relationship between TLRs and gut microbiota in terms of metabolic regulation and discuss how such interaction supports the hypothesis of intercellular energy relocation in the host, and its clinical implication in obesity and metabolic diseases.

## Metabolic regulation by TLR pathway at cellular level

### Glucose metabolism

**Fig. 1:**
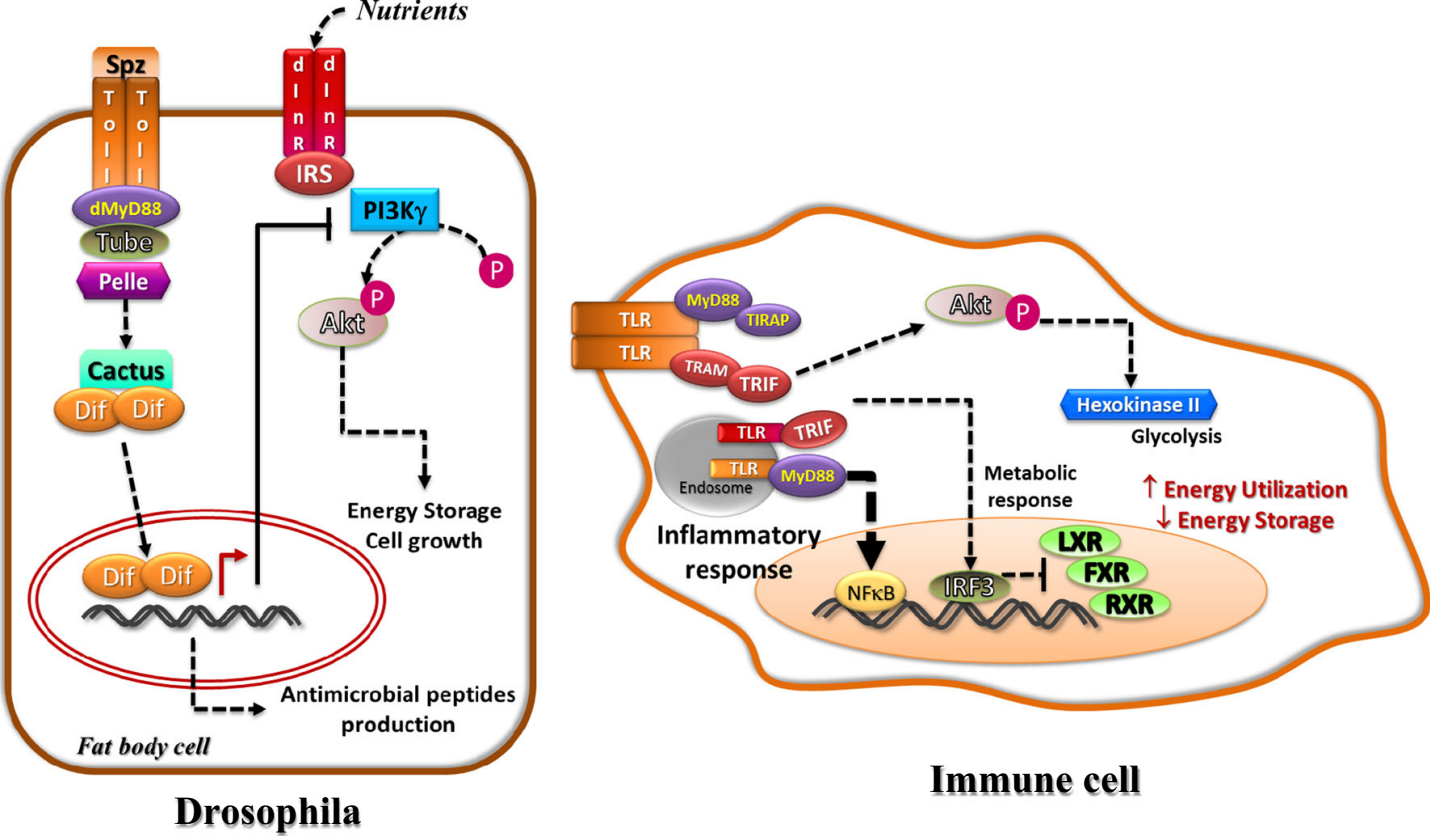
Intracellular energy relocation in fat body cell of *Drosophila* and immune cell of human. The fat body cell of *Drosophila* function as both metabolic and immune cell. Activation of toll and its adaptors, tube and dMyD88, results in the recruitment of Pelle kinase, subsequently inactivation of cactus (an orthologue of IκB), and the release of Dif (a transcription factor for antimicrobial genes). Simultaneously, insulin signaling is antagonized by the related proteins regulated by toll-induced Dif activation (*left panel*). Upon infection, activation of TLR in immune cell such as macrophage or dendritic cell stimulates inflammatory responses via the NFκB or IRF3 pathway, and IRF3 can also regulate metabolic response through interfering with the transcription activity of LXR, FXR, and RXR. In addition, phosphorylation of Akt mediated by the downstream kinases of TLR results in induction of glycolysis to generate ATP. Energy is being utilized to sustain the antimicrobial response (*right panel*)

It is well known that glycolysis plays a crucial role in macrophage polarization and dendritic cell activation. In the resting state, dendritic cells utilize lipid as their energy source through β-oxidation and oxidative phosphorylation [14, 16]. Engagement of TLR with its ligand activates the PI3K/Akt pathway and leads to a metabolic switch towards glycolysis to generate ATP [17]. Activation of the downstream kinases TBK1 and IKKε upon TLR binding induces phosphorylation of Akt, and activation of Akt triggers the enrichment of the rate-limiting enzyme for glycolysis, hexokinase-II, in the mitochondrial fraction and increases its activity [18]. During M1 macrophage polarization, a isoform switch of 6-phosphofructo-2-kinase/fructose-2,6-bisphosphatase (PFK2) from liver type (L-PFK2) to a more active ubiquitous type (u-PFK2) is observed, causing a higher glycolytic flux, and the switch is dependent on TLR (TLR2, 3, 4, and 9) pathway [19]. Conversely, during M2 polarization of macrophage in helminth infection, TLR2- and TLR4-dependent activation of MAPK cascade, and subsequently, CREB leads to IL-10 production and concurrent alteration of a series of metabolism-related genes including aconitase and ADP-dependent glucokinase [20]. Instead of aerobic glycolysis, the M2 macrophages rely on oxidative phosphorylation to utilize glucose as their energy source [20] (Fig. 1).

### Lipid metabolism

Activation of TLR3 and TLR4 in viral and bacterial infection suppresses the expression of liver X receptor (LXR)-dependent genes that regulate cholesterol efflux in macrophages [21]. The retained cholesterol acts as a reserve for the phagocytic process of macrophages [22], though in the case of atherosclerosis, it promotes foam cell formation [21, 23]. The activation of IRF3, a key downstream nuclear factor of TLR3 signaling, in response to viral infection on the one hand stimulates antiviral response through the production of interferons, and on the other hand suppresses metabolic response by downregulating retinoid X receptor-α (RXRα) [24]. RXRα can form heterodimers with other nuclear factors including peroxisome proliferator-activated receptor-γ (PPARγ), LXR, and farnesoid X receptor (FXR) which together construct a nuclear network regulating metabolism-related genes [25]. Such suppression is key to prevent viral assembly, as viruses can utilize host’s lipids to facilitate their own replication [26]. Injection of TLR3, TLR4, TLR5, TLR7, or TLR9 ligand in hepatitis B virus transgenic mice is shown to inhibit viral replication [27].

However, unlike acute response that inhibits storage and increases energy expenditure, chronic activation of TLR4 by subinfectious dose of LPS in macrophages facilitates fatty acid uptake and storage in the form of triglycerides with a parallel decrease in lipolysis and β-oxidation [28, 29]. The stimulated uptake and storage of triglycerides are also observed in adipose tissue macrophages during obesity, and the lipid accumulation is related to liposomal biogenesis [30]. The reason for a switch from glycolysis to lipid storage when low-grade inflammation sustains, and whether it is a physiological or pathophysiological phenotype is, however, unclear. It possibly serves as an adaptive mechanism to acquire an external source of energy to sustain inflammatory or antimicrobial responses, and prevent bacteria from utilizing pyruvate and acetyl-CoA for their growth, thereby limiting their growth.

## Theory of energy relocation: from immunity to obesity

Fighting against infection requires high turnover of energy [18, 31]. It is hypothesized that organisms are able to free the energy from anabolism to immunological utilization upon infection. Such energy relocation has been reported in lower rank organisms; whereas in higher rank animals and human, the findings limit to the intrinsic behaviors within immune cells (Fig. 1) [32, 33]. The “toll” in TLR was originally derived from such protein found in *Drosophila.* The functions of TLR-related molecules in these lower organisms do not limit to immunity but also include embryonic development [34]. Toll locates at the fat body cells in *Drosophila* and facilitates biosynthetic and metabolic activities [34]. The fat body is analogous to the liver and adipose tissue in human [32]. However, the fat body cells not only store excess nutrient but also synthesize hemolymph proteins, circulating metabolites as well as antimicrobial peptides [32]. In *Drosophila*, activation of toll through genetic and fungal stimulation suppresses insulin signaling pathway resulting in decreased nutrient storage and growth, and sparing of energy for the induced immunity [33]. The fat body cell is a single compartment with multiple functions, and such intrinsic metabolic regulation by toll allows an internal shift of energy utilization from usual growth and storage to immunological activities.

As the complexity of biostructure increases along the evolution of higher rank organisms, the metabolic and immune cells/organs are separated in origin. The metabolic functions of TLR pathway have been observed in immune cells only, and such regulations remain to be immunity-related in higher animals and human (see previous section). Nonetheless, TLRs are expressed in non-immune cells including adipocytes, hepatocytes, and smooth muscle cells. Although the majority of studies reported that their functions are to produce inflammatory molecules, such kind of duplicate functions seems to be redundant owing to the embedded immune cells in these tissues. In fact, a few studies have shown the metabolic functions of TLR pathway in the metabolic organs and cells. For example, activation of TLR4 by lipopolysaccharides (LPS) suppresses the expression of phosphoenolpyruvate carboxykinase (PEPCK) in the liver and adipose tissue, and the authors suggest that such decrease would result in downregulated gluconeogenesis in the liver and lipogenesis in adipose tissue [12]. TLR4 pathway is also shown to inhibit lipogenesis in muscle during fasting, and TLR4-deficient mice display a significantly higher fat mass in fasted state compared with the wild type control [35]. Moreover, treatment with LPS in rat stimulates lipolysis in adipocytes in a TLR4-dependent manner [5] (Fig. 1). These findings are consistent with the outcome of intrinsic metabolic regulation by the TLR pathway that energy is released for immunological activities rather than being stored. However, a slight decrease in body weight and adiposity with improved inflammatory status is observed in TLR4-deficient mice fed a high saturated or monosaturated fat diet, which is against the molecular mechanism of TLR4-inhibited storage of energy [36]. It is important to note that inflammation induced by immune cells in obesity aggravates insulin resistance and metabolic defects. Such dilemma is possibly because the amelioration due to suppressed inflammatory response in immune cells outweighs the sequels of the absence of TLR-mediated catabolic events in metabolic cells during long-term overnutrition in the TLR4-knockout model. By contrast, another member, TLR5, is known to play a key role in regulating colonization of gut microbiota, and its knockout model shows a drastic increase in fat mass under both normal and high-fat diet compared with wild type [1, 7]. Unlike the TLR4-deficient model, TLR5-knockout mice show a higher serum IL-1β level (i.e., pro-inflammatory status) under high-fat diet compared with wild type, which indicates an absence of immunosuppressive effect. Therefore, the inhibition of catabolism observed in these TLR5-deficient mice dominates.

In other words, the systemic metabolic phenotypes observed in these TLR-knockout models would depend on the balance between the degree of altered inflammation and the direct metabolic functions of TLRs. This balance varies among different TLRs possibly because (1) the expressions of TLRs vary in the intestine, and their blockade would thus yield the penetration of different types and amount of gut microbial products [15]. (2) The types of immunological function can be affected by the subcellular location of TLRs. For example, endosomal TLRs such as TLR3, TLR7, and TLR9 and internalization of membrane TLR4 and TLR2 into endosome result in production of type I interferons which are usually considered as anti-inflammatory mediators [37, 38]. The production of anti-inflammatory mediators along with the distinct metabolic functions of TLRs may affect the overall metabolic phenotypes. (3) The TLR-mediated metabolic functions differ in degree in the liver, adipose tissue, or muscle due to the diversity in cell types and TLR expression in these organs, and ectopic accumulation of lipids or energy away from the normal storage sites possibly aggravates metabolic dysfunction. In order to make the concept of TLR agonism/antagonism pharmacologically applicable, it is important to comprehensively investigate the expressions of TLRs in different non-immune cells and organs. For example, understanding the roles of intestinal TLRs in blocking the penetration of bacterial products would allow us to predict the availability of TLR ligands to tissues in disease states. The evaluation of metabolic and immunological capacities of TLRs in different metabolic cells using cell-specific knockout models along with in vitro studies would give us insight on generation of specific TLR agonists or antagonists for different diseases [2, 39].

## Interaction between gut microbiota and TLRs: possible intercellular energy relocation

The commensal microbes reside throughout our bodies including the skin, oral cavity, and gut, and the human gut contains 10^14^ bacteria which outnumber the total number of cells in all physiological compartments. Several TLRs have been found to affect the colonization of gut microbiota [1, 6]. For example, the first contact of gut microbiota with the intestinal lining triggers the activation of TLR5 in epithelial cells and dendritic cells, resulting in the recruitment of B cells and T cells, and subsequently the production of IgA to limit the overcolonization of gut microbiota [40]. TLR2 on T cells can sense the polysaccharide A on *Bacteroides fragilis* and control its colonization [41]. Instead of triggering major inflammation, TLRs in fact protect the host from hyperinflammation by limiting the access of bacterial products to cytosolic inflammasome, and by atypically inhibiting NFκB activation in intestinal epithelium [42, 43]. However, a study argues that the alteration of gut microbiota observed in TLR-deficient mice is due to the familial transmission attributed by housing environment or maternal transmission rather than the defects in immunity [44]. Nonetheless, regardless whether TLR has a role in alteration of gut microbiota, from the perspective of receptor-ligand dynamics, we are concerned about how much and what type of TLR ligands penetrate and what the final outcomes of TLR activation are. Certainly, we cannot rule out the possibility that change in diet or alteration of gut microbiota would also alter the expressions of TLRs, thus affecting the location of TLR-mediated catabolic events.

**Fig. 2:**
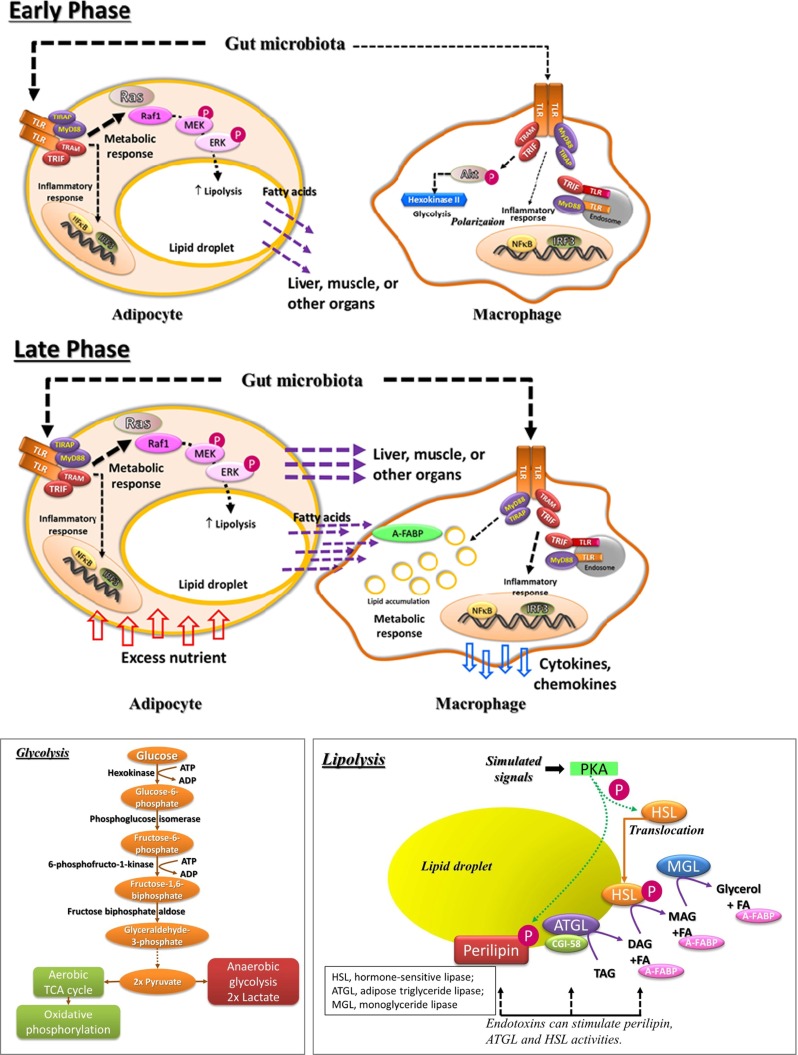
Hypothesis of intercellular energy relocation triggered by gut microbiota. Dietary alteration can result in change of gut microbiota and weakening of gut integrity, subsequently penetration of gut microbes and their products. The TLRs on metabolic cell such as adipocyte can sense the low amount of gut bacteria-derived TLR ligands, which leads to catabolic events including lipolysis. Energy or nutrient is shunted to other locations. Activation of the TLR pathway turns on glycolysis in immune cell (e.g., macrophage) to initiate polarization (*upper panel*). When the change in diet persists, e.g., overeating in obesity, other metabolic mechanisms are activated. Expansion of adipose tissue results in high turnover of lipids in adipocytes. Excess energy continuously overflows to other organs. More immune cells infiltrate into those organs, and energy or lipids are now taken up by the adjacent macrophages for their own utilization to sustain inflammation, which aggravates metabolic dysfunction (*middle panel*). The glycolysis and lipolysis pathways are briefly depicted in the boxes at the bottom (*bottom panel*)

High-fat intake or obesity can weaken the gut integrity leading to penetration of gut microbes or their products into the circulation [45]. This low-grade endotoxemia triggered by the commensal microbiota is believed to be the cause of TLR activation. Body weight change and increased adiposity are associated with alteration of gut microbiota, and the interaction between gut microbiota and TLRs can be both immunological and metabolic. Although there is yet any direct evidence showing metabolic regulation by gut microbiota-induced TLR activation, several studies have demonstrated that the metabolic functions of TLR pathway can be triggered by low-dose LPS which is similar to the range found in obesity-induced metabolic endotoxemia [5, 12]. If the concept of intercellular energy relocation holds true, immune or inflammatory response would be stimulated during nutrient overload while anabolic event would simultaneously be suppressed, which creates an insulin resistance-like condition. Changes in gut microbiota and intestinal permeability occur rapidly after initiation and termination of high-fat diet [46]. Assuming that sensitivity of TLRs is different in immune cells and metabolic cells, it is possible that the TLRs in the metabolic cells are more sensitive to the gut microbiota-derived endotoxemia, thereby metabolic alteration would be engaged first. Such metabolic regulation facilitates intercellular or inter-organ relocation of energy to prepare for the future possible full-blown attack of microbes. It would be problematic in the situation of overnutrition when the energy is not stored in the proper locations such as adipose tissue (Fig. 2). As excessive intake of energy continues, other metabolic pathways are activated. Expansion of adipose tissue results in high turnover of adipocytes as well as lipid content, and for adaptation, both catabolic and anabolic rates remain high. Excess energy continuously overflows to other organs. Our body may be misled by the sustained metabolic endotoxemia that the infection has not been subsided, resulting in infiltration of immune cells to the organs, and the immune cells in turn store the energy to sustain the inflammatory responses [28–30] (Fig. 2). In fact, it is shown that inflammation does not contribute to metabolic dysfunction in short-term high-fat diet, and the observed early-onset insulin resistance is possibly caused by lipid overload in the liver and muscle instead [47].

## Clinical implication of interfering the crosstalk between gut microbiota and TLRs

The concept of energy relocation between immunity and metabolism is based on a nutrient- or energy-scarce environment [33]. Nonetheless, the current issue faced by humankind is the long-term medical problems due to overnutrition. Obesity is often associated with metabolic endotoxemia which stimulates local and systemic inflammation and eventually aggravates metabolic dysfunction and cardiovascular risks. As a result, anti-inflammation seems to be a therapeutic option for obesity-associated diseases. However, the clinical benefit is yet conclusive, and in the case of diabetes, use of anti-inflammatory drug even increases the risk of cardiovascular event. [48] In addition, in spite of the consistent beneficial effects on metabolic functions shown in TLR-deficient animal models, no conclusive association between TLR polymorphism and metabolic diseases can be drawn from existing human data [49, 50]. A study reported that among 1894 patients without acute myocardial infarction but requiring coronary angiography, the prevalence of diabetes was 7 % lower in those with the TLR4 polymorphism (Asp299Gly) variant allele compared with the wild type allele [51]. In contrast, another study selecting a subpopulation of 722 subjects in the Cooperative Health Research in the Augsburg Region (KORA) Survey 2000 found no association with type 2 diabetes, impaired glucose tolerance, or other components of metabolic syndrome in the heterozygous and homozygous TLR4 variant alleles [50]. On the other hand, a nonsense TLR5 polymorphism prevents weight gain but imposes risk for diabetes [52]. Two studies on the association of TLR2 polymorphism (rs3804100, 1350 T/C) with type 1 diabetes showed conflicting results [53, 54]. These discrepancies may be partially related to the geographic, socioeconomic, or epigenetic influence on gut microbiota of the individuals.

Likewise, even though it is well accepted that alteration of gut microbiota can contribute to obesity and metabolic dysfunction, results from different laboratories appear to be inconsistent. The evolutionary purpose of gut microbiota is related to enhanced energy harvesting in host [55], and depletion of gut microbiota using antibiotics can promote browning of white adipose tissue and thus prevent obesity [56]. However, antibiotics have been wildly used in agriculture to stimulate weight gain of livestock in recent decades [57]. Use of antibiotics in early stage of life is associated with childhood obesity. Also, even within the same genus of *Lactobacillus*, the strain *Lactobacillus plantarum* promotes weight loss whereas *Lactobacillus ingluiviei* and *Lactobacillus acidophilus* induce weight gain [58, 59]. It is no doubt that the idea of manipulating gut microbiota to regulate body weight and metabolism requires further detailed investigation due to the complex relationship between the host and gut microbiota.

Gut microbiota pattern can be shaped by diet and substantial differences are observed in carnivores, omnivores, and herbivores [60]. Low-grade systemic inflammation induced by high-fat diet may be an evolutional protective mechanism against food-borne pathogens particularly derived from ingesting animal fat where cross infection is highly possible. In modern medicine, antagonists of TLR for metabolic and cardiovascular diseases have been explored because of the beneficial effects yielded by immunosuppression. However, if gut microbiota-derived molecules in these chronic diseases can activate TLRs, catabolism in the host would be predicted. Inhibition of the TLR pathway in such scenario would promote the energy storage; however, considering the variation in types of penetrated bacterial products and expression of TLRs in different organs and cell types, it might result in undesirable anabolic events in certain location, which would exacerbate the metabolic dysfunction. In addition, there are concerns of suppressing host TLR activity because it increases the vulnerability to infection which is also a contemporary medical issue. A thorough investigation on the functions of the TLR pathway and the interaction between TLRs and gut microbiota will allow us to better evaluate on the clinical application of agonism/antagonism of TLRs in chronic diseases.
